# Synthesis of Novel β-Keto-Enol Derivatives Tethered Pyrazole, Pyridine and Furan as New Potential Antifungal and Anti-Breast Cancer Agents

**DOI:** 10.3390/molecules201119684

**Published:** 2015-11-10

**Authors:** Smaail Radi, Said Tighadouini, Olivier Feron, Olivier Riant, Mohammed Bouakka, Redouane Benabbes, Yahia N. Mabkhot

**Affiliations:** 1 Department of Chemistry, Faculty of Sciences, University Mohamed I, Oujda-60000, Morocco; tighadouinis@gmail.com; 2 Angiogenesis and Cancer Research Lab, Pole of Pharmacology and Therapeutics-FATH5349, Institute of Experimental and Clinical Research, Université catholique de Louvain (UCL), Brussels 1200, Belgium; olivier.feron@uclouvain.be; 3 Molecules, Solids and Reactivity (MOST), Institute of Condensed Mater and Nanosciences (IMCN), Université catholique de Louvain (UCL), Place Louis Pasteur 1, Louvain-la-Neuve 1348, Belgium; olivier.riant@uclouvain.be; 4 Department of Biologie, Faculty of Sciences, University Mohamed I, Oujda-60000, Morocco; bouakkam@yahoo.fr (M.B.); red.bes72@gmail.com (R.B.); 5 Department of Chemistry, Faculty of Science, King Saud University, P. O. Box 2455, Riyadh 11451, Saudi Arabia; yahia@ksu.edu.sa

**Keywords:** keto-enols, heterocycles, breast cancer, fungal strains

## Abstract

Recently, a new generation of highly promising inhibitors bearing β-keto-enol functionality has emerged. Reported herein is the first synthesis and use of novel designed drugs based on the β-keto-enol group embedded with heterocyclic moieties such as pyrazole, pyridine, and furan, prepared in a one-step procedure by mixed Claisen condensation. All the newly synthesized compounds were characterized by FT-IR, ^1^H-NMR, ^13^C-NMR, ESI/LC-MS, elemental analysis, and evaluated for their *in vitro* antiproliferative activity against breast cancer (MDA-MB241) human cell lines and fungal strains (*Fusarium oxysporum f.sp albedinis FAO*). Three of the synthesized compounds showed potent activity against fungal strains with IC_50_ values in the range of 0.055–0.092 µM. The results revealed that these compounds showed better IC_50_ values while compared with positive controls.

## 1. Introduction

**Figure 1:**
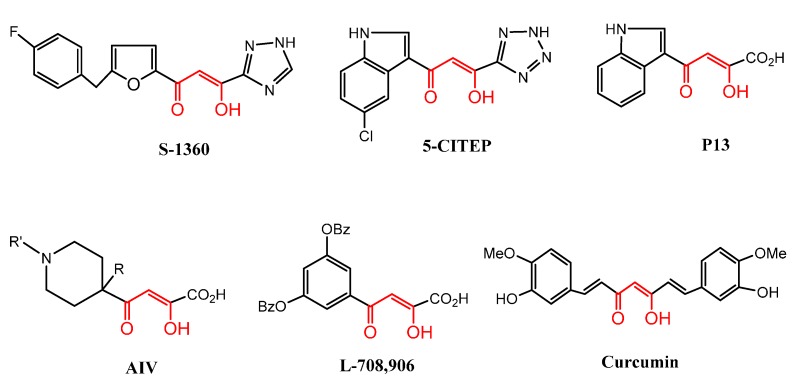
Representative drugs containing keto-enol functionality.

Heterocyclic compounds with β-keto-enol moieties are well established as important, biologically effective compounds. Their versatile utility in the world of medicinal chemistry is firmly established [1,2]. Among this class of drug, we cite anti-HIV drugs S-1360 (Shionogi, Ltd., Florham Park, NJ, USA) [3] and L-708,906 (Merck Research Laboratories, Boston, MA, USA) [4] in clinical trials as well as P13 (IC_50_ = 1.1 µM) [5], the 5-CITEP designed by the National Cancer Institute (Bethesda, MD, USA) [3], and AIV(IC_50_ = 0.3 µM), an inhibitor of anti-influenza virus [6] (Figure 1), *etc*.

In this context, we note also the interest in curcumin [7] derivatives with β-keto-enol pharmacophore sites (Figure 1) which have spurred numerous studies in medicinal chemistry owing to their capability of eliciting antioxidant [8], anti-HIV [9], antitumor [10,11,12], and anti-inflammatory [13] activities.

The β-keto-enol pharmacophore site of these hybrid drug molecules has the potential advantage of being active against all genotypes of the virus and drug-resistant variants. Multistage processes may explain the biological responses of this motif (β-keto-enol), such as (i) its penetration into blood vessel walls and plasma cell membranes; (ii) its interaction with the active site; (iii) its ability to chelate with metals in biological processes; and (iv) its reaction with oxygen (under aerobic conditions) or with cell macromolecules (in hypoxic conditions) resulting in oxidative stress, the modulation of gene expression, and a complex immune response to hapten-conjugate adducts.

Consequently, significant effort is devoted to the search for drug-like scaffolds bearing the β-keto-enol pharmacophore. Recently, several molecules were designed, such as the calix[4]arene derivatives, containing the triazolyl keto-enol moiety showing potent integrase strand transfer inhibitory activity [14], keto-enol tetrazoles, and triazoles as anti-HCV agents [15], and coumarinyl chalcones, exhibiting high selectivity for the breast cancer cell lines [16].

Accordingly, described herein is the first synthesis and examination of some hybrid drug molecules bearing the β-keto-enol functionality as a useful motif, especially in fungal and cancer activities.

## 2. Results and Discussion

### 2.1. Chemistry

**Scheme 1:**
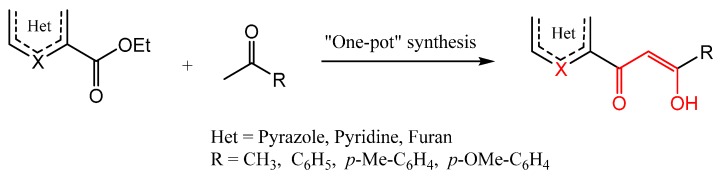
Reagents and conditions: Na, Toluene, rt, two days, then acetic acid.

**Scheme 2:**
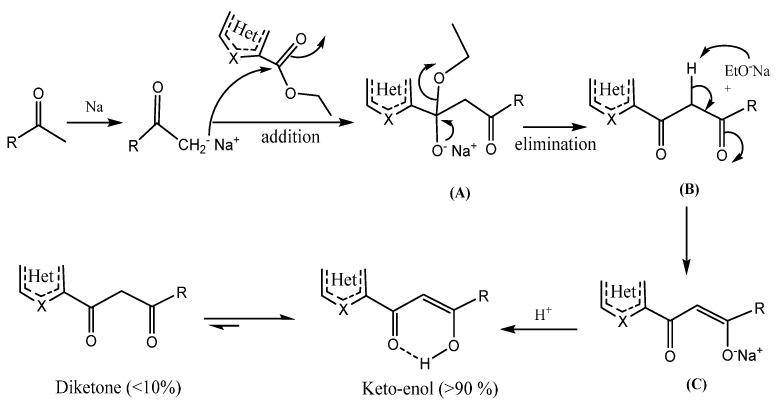
Proposed mechanism of the formed products.

The target compounds based on β-keto-enol group-tethered pyrazole, pyridine, and furan were prepared by a one-pot *in situ* condensation as illustrated and outlined in Scheme 1 and Scheme 2.

The sodium metal-mediated condensation of ketone derivatives with ethyl heterocycle-2-carboxylates exclusively afforded the target products in their enol tautomeric form. The reaction, as a mixed Claisen condensation, was carried out under mild conditions (room temperature, two days), using toluene as a solvent and sodium metal as the base. The reaction was slow and gave better results (with respect to the purity of the compounds).

The synthesis began with the formation of a ketone enolate nucleophile in cool conditions (0 °C). After adding the appropriate heterocyclic carboxylate, the resulting mixture was stirred at room temperature for two days. The formed enolate initially underwent nucleophilic attack at the ester carbonyl to produce tetrahedral intermediate (**A**).

The expulsion of the ethoxide ion from the unstable tetrahedral intermediate of the initial Claisen adduct yielded a β-diketone (**B**). The expelled base (EtO^−^Na^+^) then removed an acidic alpha proton from the β-diketone to generate a stabilized enolate ion product (**C**) as a precipitated salt. This formed precipitate was filtered, washed with toluene, dissolved in water, and neutralized with acetic acid to pH = 5 to afford the title products in acceptable yield after being filtered through silica using CH_2_Cl_2_/MeOH as an eluant. The mechanism for the formation of these target products is given in Scheme 2.

It is emphasized that our products exist exclusively in “3-hydroxy-alk-2-en-1-one” form as confirmed by the spectral data; these tautomeric forms were also confirmed by XRD and the results will appear in due course [17]. However, two products (similar to **5** and **6**) were obtained in previous work by others, in the “4-hydroxy-alk-3-en-2-one” tautomeric form under different conditions (NaH, Et_2_O, reflux) [18].

The β-keto-enol form is greatly favored over the β-diketone form because of the conjugation of the enol with the carbonyl group, and the stability gained, by a strong six-centered intramolecular hydrogen bond. The β-keto-enol interconversion rate (>90%) was determined using the ^1^H-NMR integration of signals from the enol =C-H and the ketone CH_2_. Indeed, according to the NMR spectra, the parent β-diketones exist almost exclusively in the enol form and only a trace of the keto form is seen around 4 ppm. In DEPT-135, a very small negative signal from CH_2_ was also observed. Finally, crystals of most of the β-keto-enols were isolated from methanol by slow evaporation. Compound **6** was recently reported in a different way [19].

### 2.2. Biological Activities

All synthesized β-keto-enol heterocycles were evaluated for their activity against breast cancer (MDA-MB241) human cell lines using normoxic conditions [20], and against fungal strains (*Fusarium oxysporum f.sp albedinis FAO*) using the agar diffusion technique (ADT) [21]. It is of note that all products were also tested against three bacterial strains (*Echerichia coli*, *Bacillus subtilis*, and *Micrococcus luteus*), but no significant effect was observed against these organisms.

**Table 1:** Breast cancer and fungal inhibitory activities of synthesized heterocycle β-keto-enols.
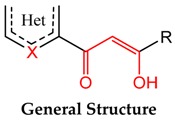

Products			MDA-MB241		*Fusarium Oxysporum f.sp Albedinis*	
No.	Heterocycles	R	IC_50_ (μg/mL)	IC_50_ (μM)	IC_50_ (μg/mL)	IC_50_ (μM)
**1**	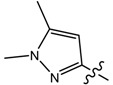	CH_3_	46.20	256.38	0.01	0.055
**2**	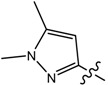	C_6_H_5_	44.33	183.00	12.83	53.39
**3**	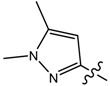	*p*-Me-C_6_H_4_	21.95	85.65	142	554.03
**4**	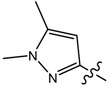	*p*-MeO-C_6_H_4_	34.93	128.30	150	550.86
**5**	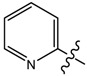	CH_3_	47.00	288.04	0.013	0.079
**6**	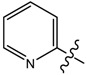	C_6_H_5_	17.62	78.23	16.43	72.94
**7**	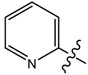	*p*-Me-C_6_H_4_	128.67	537.8	35.80	149.62
**8**	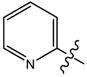	*p*-MeO-C_6_H_4_	28.97	113.50	N/A *	N/A *
**9**	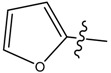	CH_3_	N/A *	N/A *	0.014	0.092
**10**	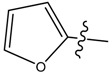	C_6_H_5_	18.79	87.72	68 .45	319.53

* N/A means non applicable because IC_50_ > 100 µg/mL.

However, the results of the anticancer and antifungal effects were very significant and are given in Table 1, respectively. Most of these molecules were cytotoxic against breast cancer cell lines in a dose-dependent manner. The activity followed the structure activity relationships (SARs) and showed an interesting dependence on the substitution pattern. Considering the influence of substituent R, it was found that the phenyl residue leads to a stronger growth inhibition [18]. This was especially evident for products **6** and **10**. The concentration required to induce the activity (IC_50_) was more pronounced for compounds **3**, **6** and **10** with IC_50_ values of 21.95, 17.62, and 18.79 μg/mL, respectively. Beside this observation, we also noted the effect of the heterocycle groups for appreciable biological activity.

These structures have also led to unexpected antifungal activity. Indeed, compounds **1**, **5**, and **9** with methyl in the R position had the most potent activity with IC_50_ values of 0.055, 0.079, and 0.092 μM, respectively. This result was better than all the described products. We noted that the substitution of methyl in the R position was essential for this biological activity. The aryl groups strongly decreased the activity. This suggests that the marked bioactivity of the heterocyclic compounds was sensitive to modifications and could be further exploited to determine the structure activity relationship around this novel class of fungal inhibitors. Other structural modifications to these active compounds as antifungal and anti-HIV candidates are currently in progress.

## 3. Experimental

### 3.1. General Information

All commercial reagents were analytical grade (Aldrich, purity >99%, St. Louis, MO, USA). Melting points were measured using a BUCHÏ 510 m.p. apparatus (Oujda, Morocco). ^1^H- and ^13^C-NMR spectra were performed on a Bruker AC 300 spectrometer (CNRS, Rabat, Morocco) (300 MHz for ^1^H and 75.47 MHz for ^13^C spectra). JEOL JMS DX-300 mass spectrometer (Rabat, Morocco) was used for the determination of molecular weights. Infrared (IR) spectra were recorded on a Shimadzu infrared spectrophotometer (Oujda, Morocco) using the KBr disc technique. The formazan obtained at the end of the experiment in MTT assays was measured by means of a Perkin Elmer Victor X4 Microplate reader (Brussels, Belgium).

### 3.2. General Procedure for the Synthesis of β-Keto-Enol Heterocycles

To a suspension of sodium (15.21 mmol) in 20 mL of toluene, the appropriate heterocyclic carboxylate (12.01 mmol) in 25 mL of toluene was slowly added; then acetone or aryl methyl ketones (12.01 mmol) in 10 mL of toluene was added at 0 °C. The resulting mixture was stirred at room temperature for two days. The precipitate formed was filtered, washed with toluene, dissolved in water, and neutralized with acetic acid to pH 5. After extraction with CH_2_Cl_2_, the organic layer was dried over anhydrous sodium sulfate and concentrated in *vacuo*. The obtained residue was filtered through silica using CH_2_Cl_2_/MeOH as eluant to give the desired products **1**–**10** as a white solid in 35%–48% yield. β-keto-enol forms were recrystallized from methanol (95%) to obtain target compounds **1**–**10** which were confirmed by FT-IR, ^1^H-NMR, ^13^C-NMR, elemental analysis, and mass spectroscopy.

*(Z)-1-(1.5-Dimethyl-1H-pyrazol-3-yl)-3-hydroxybut-2-en-1-one* (**1**). Yellow crystal; yield: 35%; m.p. 83–84 °C; *R_f_* = 0.66 (diethyl ether)/silica. IR (KBr, cm^−1^): ν (OH) = 3422; ν (C=O) = 1619; ν (enolic C=C) = 1509; ^1^H-NMR (CDCl_3_): δ 2.11 (s, 3H, -CH_3_); 2.27 (s, 3H, Pz-CH_3_); 3.83 (s, 3H, N-CH_3_); 6.29 (s, 1H, enol, C-H). 6.54 (s, 1H, Pz-H); ^13^C-NMR (CDCl_3_): δ 11.2 (1C, Pz-CH_3_); 24.6 (1C, CH_3_); 36.7 (1C, N-CH_3_); 96.7 (1C, enol C-H); 106.1 (1C,Pz-CH) 140.5 (1C, Pz, C-CH_3_); 146.9 (1C, C=N); 180.4 (1C, C=O); 190.0 (C-OH); MS: *m*/*z*, 181.00 (M + H)^+^. Anal. Cald. for C_9_H_12_N_2_O_2_: C, 59.99; H, 6.71; N, 15.55. Found: C, 60.11; H, 6.83; N, 15.43.

*(Z)-1-(1,5-Dimethyl-1H-pyrazol-3-yl)-3-hydroxy-3-phenylprop-2-en-1-one* (**2**). Yellow crystal; yield: 32%; m.p. 108–110 °C; *R_f_*: 0.88 (CH_2_Cl_2_/MeOH 9/1/silica), IR (KBr, cm^−1^): ν (OH) = 3416; ν (C=O) = 1639; ν (enolic C=C) = 1518; ^1^H-NMR (DMSO-*d*_6_): δ 2.29 (s, 3H, Pz-CH_3_); 3.83 (s, 3H, N–CH_3_); 6.60 (s, 1H, enol, C-H); 7.02 (s, 1H, Pz-H); 7.58 (m, 3H, Ar-H_4,3,5_); 7.95 (m, 2H, Ar-H_2,6_). ^13^C-NMR (DMSO-*d*_6_): δ 11.9 (1C, Pz-CH_3_); 37.3 (1C, CH_3_-N); 93.3 (1C, enol C-H); 106.1 (1C, CH-Pz); 127.3 (2C, Ar-C_2,6_); 128.8 (1C, Ar-C_4_); 129.3 (2C, Ar-C_3,5_); 148.8 (1C, Pz, C-CH_3_); 181.7 (1C, C-OH); 183.5 (1C, C=O); MS: *m*/*z*, 243.10 (M + H)^+^.

*(Z)-1-(1,5-Dimethyl-1H-pyrazol-3-yl)-3-hydroxy-3-p-tolyprop-2-en-1-one* (**3**). Yellow crystal; yield: 22%; m.p. 132–134 °C; *R_f_*: 0.29 (diethyl ether)/silica. IR (KBr, cm^−1^): ν (OH) = 3412; ν (C=O) = 1642; ν (enolic C=C) = 1536; ^1^H-NMR (DMSO-*d*_6_): δ 2.26 (s, 3H, *p*-CH_3_-Ar); 2.36 (s, 3H, Pz-CH_3_); 3.82 (s, 3H, CH_3_-N); 6.67 (d, 1H, Pz-H); 6.98 (s, 1H, enol, C-H); 7.32 (m, 2H, Ar-H_3,5_); 7.85 (m, 2H, Ar-H_2,6_). ^13^C-NMR (DMSO-*d*_6_): δ 11.2 (1C, Pz-CH_3_); 21.6 (1C, *p-*CH3-Ar); 37.3 (1C, CH_3_-N); 92.8 (1C, enol C-H); 106.0 (1C, CH-Pz); 128.1 (2C, Ar-C_2,6_); 129.9 (2C, Ar-C_3,5_); 182.2 (1C, C-OH); 182.9 (1C, C=O); MS: *m*/*z*, 257.11 (M + H)^+^.

*(Z)-1-(1,5-Dimethyl-1H-pyrazol-3-yl)-3-hydroxy-3-(4-methoxyphenyl)prop-2-en-1-one* (**4**). Yellow crystal; yield: 31%; m.p. 122–124 °C; *R_f_*: 0.61 (CH_2_Cl_2_/MeOH 8/2)/silica. IR (KBr, cm^−1^): ν (OH) = 3432; ν (C=O) = 1678; ν (enolic C=C) = 1528; ^1^H-NMR (DMSO-*d*_6_): δ 2.26 (s, 3H, O-CH_3_); 3.82 (s, 3H, Pz-CH_3_); 3.83 (s, 3H, CH_3_-N): 6.65 (s, 1H, Pz-H); 6.94 (s, 1H, enol, C-H); 7.04 (m, 2H, Ar-H_3,5_); 7.94 (m, 2H, Ar-H_2,6_); ^13^C-NMR (DMSO-*d*_6_): δ 11.1 (1C, Pz-CH_3_); 37.3 (1C, CH_3_-N); 56.0 (1C, *p*-OCH3-Ar); 92.2 (1C, enol C-H); 106.1 (1C, =CH-Pz); 114.6 (2C, Ar-C_3,5_); 129.6 (2C, Ar-C_2,6_); 181.7 (1C, C-OH); 182.9 (1C, C=O); MS: *m*/*z*, 273.06 (M + H)^+^.

*(Z)-3-Hydroxy-1-(pyridin-2-yl)but-2-en-1-one* (**5**). Brown powder; yield: 48%; m.p. = 59–61 °C; *R_f_*: 0.27 (CH_2_Cl_2_/MeOH 9/1)/silica. IR (KBr, cm^−1^): ν (OH) = 3448; ν (C=O) = 1611; ν (enolic C=C) = 1565; ^1^H-NMR (CDCl_3_): δ 2.23 (s, 3H, -CH_3_); 6.94 (s, 1H, enol, C-H); 7.42 (t, 1H, Py-H_β_); 7.85 (t, 1H, Py-H_γ_); 8.08 (d, 1H, Py-H_δ_); 8.66 (d, 1H, Py-H_ᾳ_); ^13^C-NMR (CDCl_3_): δ 26.2 (1C, CH_3_-C=O); 97.8 (1C, enol, CH); 123.0 (1C, Py-C_δ_); 126.4 (1C, Py-C_β_); 138.0 (1C, Py-C_γ_); 148.5 (1C, Py-C_ᾳ_); 151.4 (1C, Py-C_ε_); 179.3 (1C, C-O), 195.6 (1C, C=OH); MS: *m/z*, 164.07 (M + H)^+^. Anal. Calcd. for C_9_H_9_NO_2_: C, 66.25, H, 5.56; N, 8.58. Found: C, 66.31; H, 5.62, N, 5.40.

*(Z)-3-Hydroxy-3-phenyl-1-(pyridin-2-yl)prop-2-en-1-one* (**6**). Red powder; yield: 32%; m.p. = 78–80 °C; *R_f_*: 0.54 (CH_2_Cl_2_/MeOH 9/1)/silica. IR (KBr, cm^−1^): ν (OH) = 3438; ν (C=O) = 1600; ν (enolic C=C) = 1549; ^1^H-NMR (DMSO-*d*_6_): δ 7.51 (s, 1H, enol CH); 7.61 (m, 3H, Ar-H_4,3,5_); 7.66 (d, 2H, Ar-H_2,6_); 8.00 (t, 1H, Py-H_β_); 8.04 (t, 1H, Py-H_γ_); 8.11 (d, 1H, Py-H_δ_); 8.77 (d, 1H, Py-H_ᾳ_). ^13^C-NMR (DMSO-*d*_6_): δ 93.6 (1C, enol C-H); 122.5 (1C, Py-C_δ_); 127.7 (2C, Ar-C_2,6_); 128.8 (1C, Ar-C_4_); 129.4 (2C, Ar-C_3,5_); 133.7 (1C, Py-C_β_); 138.3 (1C, Py-C_γ_); 150.1 (1C, Py-C_ᾳ_); 184.4 (1C, C-OH); 186.1 (1C, C=O); MS: *m*/*z*, 226.13 (M + H)^+^. Anal. Calcd. for C_14_H_11_NO_2_: C, 74.65; H, 4.92; N, 6.22. Found: C, 74.74, H, 4.89; N, 6.25.

*(Z)-3-Hydroxy-1-(pyridin-2-yl)-3-p-tolylprop-2-en-1-one* (**7**). Brown powder, yield 27%, m.p.: 174–176 °C; *R_f_*: 0.75 (CH_2_Cl_2_/MeOH 6/4)/silica. IR (KBr, cm^−1^): ν (OH) = 3444; ν (C=O) = 1602; ν (enolic C=C) = 1542; ^1^H-NMR (DMSO-*d*_6_): δ 2.49 (s, 3H, *p*-CH_3_-Ar); 6.78 (s, 1H, enol CH); 7.31 (d, 2H, Ar-H_3,5_); 7.46 (d, 2H, Ar-H_2,6_); 7.91 (dd, 2H, Py-H_β,γ_); 8.12 (d; 1H, Py-H_δ_); 8.89 (d, 1H, Py-H_ᾳ_). ^13^C-NMR (DMSO-*d*_6_): δ 21.3 (1C, *p*-CH_3_-Ar); 93.8 (1C, enol CH); 121.7 (1C, Py-H_δ_); 126.1 (2C, Ar-C_2,6_); 128.1 (1C, Py-H_β_); 128.9 (2C, Ar-C_3,5_); 136.1 (1C, Py-H_γ_); 149.6 (1C, Py-H_ᾳ_); 178.1 (1C, C-OH); 186.1 (1C, C=O); MS: *m*/*z*, 240.13 (M + H)^+^. Anal. Calcd. for C_15_H_13_NO_2_: C, 75.30; H, 5.48, N, 5.85. Found: C, 75.49; H, 5.44; N, 5.80.

*(Z)-3-Hydroxy-3-(4-methoxyphenyl)-1-(pyridin-2-yl)prop-2-en-1-one* (**8**). Brown clear crystal, yield 33%, m.p.: 112–114 °C; *R_f_*: 0.33 (CH_2_Cl_2_/MeOH 9/1)/silica. IR (KBr, cm^−1^): ν (OH) = 3444; ν (C=O) = 1599; ν (enolic C=C) = 1549; ^1^H-NMR (DMSO-*d*_6_): δ 3.83 (s, 3H, O-CH_3_); 7.08 (d, 2H, Ar-H_3,5_); 7.50 (s, 1H, enol, CH); 7.62 (t, 1H, Py-H_δ_); 7.97(t, 1H, Py-H_β_) 8.04 (d, 2H, Ar-H_2,6_); 8.08 (t, 1H, Py-H_γ_); 8.76 (d, 1H, Py-H_ᾳ_). ^13^C-NMR (DMSO-*d*_6_): δ 56.1 (1C, OCH_3_); 92.8 (1C, enol, CH); 114.5 (2C, Ar-C_3,5_); 122.2 (1C, Py-C_δ_); 127.5 (2C, Ar-C_2,6_); 138.2 (1C, Py-C_γ_); 150.1 (1C, Py-C_ᾳ_); 182.1 (1C, C-OH); 186.6 (1C, C=O); MS: *m*/*z*, 256.08 (M + H)^+^. Anal. Calcd. for C_15_H_13_NO_3_: C, 70.58; H, 5.13; N, 5.49. Found: C, 70.76; H, 5.20, N, 5.59.

*(Z)-1-(Furan-2-yl)-3-hydroxybut-2-en-1-one* (**9**). Red hygroscopic; yield: 39%; *R_f_*: 0.91 (CH_2_Cl_2_/MeOH 8/2)/silica. IR (KBr, cm^−1^): ν (OH) = 3434 cm^−1^; ν (C=O) = 1620 cm^−1^; ν (enolic C=C) = 1468; ^1^H-NMR (CDCl_3_): δ 2.13 (s, 3H, -CH_3_); 6.06 (s, 1H, enol, C-H); 6.52 (m, 1H, Fu-H_β_); 7.14 (d, 1H, Fu-H_γ_); 7.55 (m, 1H, Fu-H_ᾳ_); ^13^C-NMR (CDCl_3_): δ 24.4 (1C, CH_3_-C=O); 96.0 (1C, C-H, enol); 112.4 (1C, Fu-C_γ_); 115.5 (1C, Fu-C_β_); 145.9 (1C, Fu-C_ᾳ_); 150.0 (1C, Fu-C_δ_); 176.1 (1C, C=O), 19.5 (1C, C-OH); MS: *m*/*z*, 153.10 (M + H)^+^. Anal. Calcd. for C_8_H_8_O_3_: C, 63.15; H, 5.30. Found: C, 63.25, H, 5.38.

*(Z)-1-(Furan-2-yl)-3-hydroxy-3-phenylprop-2-en-one* (**10**). Red powder; yield 42%; m.p. 64–66 °C; *R_f_*: 0.61 (CH_2_Cl_2_/silica). IR (KBr, cm^−1^): ν (OH) =3431; ν (C=O) = 1621; ν (enolic C=C) = 1531; ^1^H-NMR (DMSO-*d*_6_): δ 6.72 (d, 1H, Fu-H_γ_); 7.01 (s, 1H, enol, C-H); 7.52 (d, 2H, Ar-H_3,5_); 7.58 (t, 1H, Ar-H_4_); 7.64 (d, 2H, Ar-H_2,6_); 8.00 (t, 1H, Fu-H_β_); 8.04 (d, 1H, Fu-H_ᾳ_); ^13^C-NMR (DMSO-*d*_6_): δ 93.3 (1C, C-H, enol); 113.5 (1C, Fu-C_γ_); 120.2 (1C, Fu-C_β_); 127.3 (2C, Ar-C_3,5_); 129.2 (3C, Ar-C_2,4,6_); 148.7 (1C, Fu-C_ᾳ_); 178.5 (1C, C=O); 180.8 (1C, C-OH); MS: *m*/*z*, 215.12 (M + H)^+^.

### 3.3. Anticancer Assays

Prepared compounds were screened against breast cancer (MDA-MB241) human cell lines using normoxic conditions [20]. Tests were performed in Angiogenesis and Cancer Research Lab, Institute of Experimental and Clinical Research (UCL, Brussels, Belgium).

### 3.4. Antibacterial and Antifungal Tests

The *in vitro* antifungal activities were tested by the agar diffusion technique [21] using fungal strains (*Fusarium oxysporum f.sp albedinis FAO*).

The results were compared with positive controls (benomyl and thiophanate-methyl).The *in vitro* antibacterial activities were tested using bacterial strains (*Echerichia coli*, *Bacillus subtilis*, and *Micrococcus luteus*).

## 4. Conclusions

In summary, we have described the first synthesis of novel β-keto-enols embedded with heterocyclic moieties and the evaluation of their *in vitro* anticancer and antifungal activities. Most of the compounds showed modest antiproliferative activity against breast cancer (MDA-MB241) human cell lines. Among the synthesized products, compounds **1**, **5**, and **9** successfully showed the most potent antifungal activity with IC_50_ values in the range of 0.055–0.092 μM as compared with positive controls.
